# Knowledge, Attitude, and Risk Perception in Oral Isotretinoin Use: A Cross-Sectional Study from Jordan

**DOI:** 10.1155/2024/7714527

**Published:** 2024-06-15

**Authors:** Mai I. Al-Hawamdeh, Mariam Al-Ameri, Salli Lutfi, Nidal Muhtaseb, Rasha Takhayneh, Tasneem Awamreh

**Affiliations:** ^1^College of Pharmacy, Department of Pharmacy, Amman Arab University, Amman, Jordan; ^2^Faculty of Pharmacy, Department of Clinical Pharmacy and Pharmacy Practice, Yarmouk University, Irbid, Jordan

## Abstract

The most prevalent skin condition is acne vulgaris. Recent clinical practice guidelines recommend oral isotretinoin to treat moderate-to-severe acne. The aim of this study is to assess the knowledge, attitude, and risk perception of oral isotretinoin for acne treatment. This is a cross-sectional descriptive study conducted in the country of Jordan. The study sample includes people resident in Jordan aged ≥14 years who have been treated with oral isotretinoin for acne. The study involved 373 participants who previously used oral isotretinoin for skin disorders. Most were Jordanian (89.3%), aged 19–25 (37.3%), and from the central region (82.8%). Mostly, they used isotretinoin for severe or mild acne (25.2% and 24.1%, respectively), rosacea (4.1%), or to alleviate acne scars. Surprisingly, 58.1% did not consult their specialist for side effects, and 20% shared their treatment. The average proper use score was 9.98 out of 16. A link was found between higher risk knowledge scores and proper use scores. Side effects such as nausea, irregular heartbeat, and pancreatitis affected some users (11.5%, 10.5%, 7.0%, and 3.2%, respectively). Knowledge about isotretinoin's risks varied, with percentages recognizing teratogenicity (57.7%), liver damage (52.6%), and lipid profile effects (37.2%), while 25% believed that they had no side effects. The study revealed partial adherence to oral isotretinoin guidelines, with gaps in monitoring and consultation. A positive correlation emerged between risk knowledge and proper usage, emphasizing the need for comprehensive education and monitoring strategies in isotretinoin therapy for skin disorders.

## 1. Introduction

The most prevalent skin condition is acne vulgaris, which is a persistent, multifactorial, inflammatory condition that affects the sebaceous glands, ducts, and hair follicles. It frequently affects the face, back, and chest [1]. Acne vulgaris places a heavy burden on the sufferer due to the worry, low self-esteem, stigma, and face scarring that are all connected with it [2]. Males are more likely to get acne vulgaris throughout adolescence than females, although females are more likely to develop the condition later in adulthood [2, 3]. Patients with acne vulgaris may experience psychosocial effects, and female patients are more likely to experience these effects than male patients [4]. Oral isotretinoin is often administered as a first-line treatment for severe nodulocystic and papulopustular acne, which may cure the condition [5].

The Food and Drug Administration (FDA) approved 13-cis-retinoic acid, also known as isotretinoin, as a pharmacological treatment for acne vulgaris in 1982 [6]. Isotretinoin was recommended for moderate-to-severe nodulocystic acne that had shown resistance to prior medication regimens [7]. In 1997, Cunliffe et al. also advised the use of isotretinoin for mild cases. Despite the demonstrated efficacy of isotretinoin, its precise mechanism of action remains unknown. It is hypothesized that isotretinoin impacts sebaceous glands by inducing apoptosis, which has a comparable effect on skin lipid synthesis and other systems [8]. Recent clinical practice guidelines from Canada, the United States, and Europe recommend isotretinoin should be used to treat moderate-to-severe acne [9, 10].

Oral isotretinoin is authorized for the use as first-line therapy for the treatment of moderate or sever acne that does not improve with other medications [11]. Moreover, fatal adverse effects are extremely rare, and to now, there is not any report of death which proved to be related to oral isotretinoin [12]. Clinically significant side effects of isotretinoin fall into two main categories: mucocutaneous, which include cheilitis and face dermatitis, and systemic, which includes teratogenicity [13]. Other possible side effects include nosebleeds, dizziness, eye irritation, joint and back discomfort, depression, dyslipidaemia, dryness, and altered liver function [14].

The poor safety record of isotretinoin therapy highlights the need to increase patient knowledge of the medication and the best ways to use it. The current study's aim is to assess the knowledge, attitude, and risk perception in oral isotretinoin use among Jordanian population. The results of this study should enhance our understanding of the local situation, contribute to the development of more effective interventions to increase patient awareness and knowledge about isotretinoin use, and ultimately improve safety and health outcomes for isotretinoin users.

## 2. Method

This is a cross-sectional study conducted from March 1st to the end of May 2023 within the geographical boundaries of Jordan utilizing a Google Form survey. The study population includes male or female participants resident in Jordan who are 14 years of age or older and have previously used oral isotretinoin dosage form for the treatment of acne skin condition.

The convenience sampling method was used in this study for data collection. A web-based self-administered survey was created using Google Forms and distributed randomly via e-mail and social media platforms such as WhatsApp, Instagram, and Facebook. The survey was written and distributed in Arabic language with medical terminology being simplified to the public using public terms. Participants were given clarification on the active ingredient “Isotretinoin” since they were more familiar with trade names rather than the active and medical terms or ambiguous words, such as pattern of use, medical conditions, and side effects.

The survey consists of three sections. The first section collected demographic data, including age, gender, academic degree, marital status, and place of residency in Jordan. The rest of the survey included questions that were intentionally not divided into separate sections for the purpose of avoiding participants from being led to the correct practice. First part of the questions was related to participants' practices regarding oral isotretinoin administration such as used doses, adherence to treatment course, performing suitable lab analysis, and the taken precautionary measures to avoid possible side effects while the other part assesses the misuse of isotretinoin indirectly by asking about sharing the medications with other patients, using expired products, getting the medication without prescription, referring back to medical specialist and the appearance of uncommon side effects.

Initial draft of the survey was built after reviewing the literature. Content and face validity were performed by pharmacists and university instructors by evaluating the relevancy, clarity, spelling, and comprehension of different parts of the survey. A pilot study involving a minimum of 10 subjects was undertaken to test the reliability of the final draft of the survey; internal reliability was assessed by Cronbach's alpha coefficient (*α* = 0.72). An introductory paragraph was created, which insured the confidentiality and anonymity of data collection and analysis. Regarding consenting the participants, the questionnaire contained a statement “I consent to participate in the study” after the introductory paragraph, which the participant must click before proceeding to the first question.

Sample size calculation was based on published literature; a convenience sample size design was used [10, 15]. For this observational study, the following equation for an unlimited population will be used to determine the sample size:

(1) $$\begin{array}{c} N=\frac{\left[{\left(z_{\alpha /2}\right)}^{2}*P^{*}\left(1-P\right)\right]}{d^{2\left[15\right]}}, \end{array}$$

where *n* is the sample size, *Z* is the statistic corresponding to the level of confidence, for a 95% confidence interval *Z* is 1.96, *P* is expected prevalence (results from similar studies), and *d* is precision, *d* = 0.05. The prevalence of acne vulgaris among adolescents and young adults in Jordan is 45% [16]. This equation results in the largest sample size when *P* = 0.45 and 1 − *P* is 0.55. By using the above values, the sample size is 380 participants.

The study was reviewed and approved by the Institutional Review Board at Amman Arab University, Deanship of Scientific Research. Informed consent was included as the first part of the survey and had been obtained from each participant prior to participation. No data were saved before the participants submitted their complete answers, and the participants were free to leave the study at any time without providing a reason.

Statistical tests were performed using the Statistical Package for the Social Sciences (SPSS) version 27.0 (SPSS Inc. Chicago, IL, USA, 2020). Initially, descriptive statistics were conducted for demographic variables, the use and misuse of study medications. All data were represented by frequencies and percentages. The associations between different demographic parameters and the attitude score were analysed using unpaired *T*-test and ANOVA, with a confidence level of 95% and a significance value of *p* ≤ 0.05.

## 3. Results

A total of 373 subjects who had previously used oral isotretinoin for the treatment of skin disorders participated in the study and successfully completed the survey. The majority of participants were Jordanian (*n* = 333, 89.3%), aged between 19 and 25 years (*n* = 139, 37.3%), female (*n* = 285, 76.4%), and residing in the central region (*n* = 309, 82.8%). Regarding their level of education, 68.1% of the participants had completed their bachelor's degree. Detailed demographic data are presented in Table 1.

**Figure 1:**
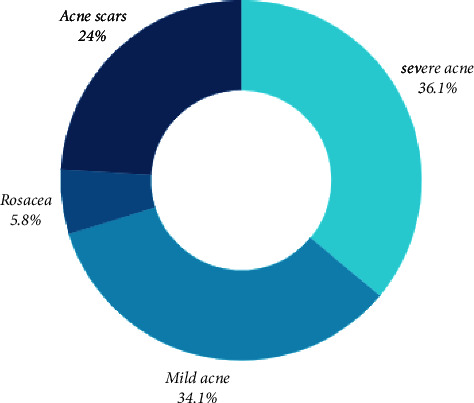
Participants' rationale for utilizing oral isotretinoin therapy.

As for the reason for using oral isotretinoin treatment, 25.2% of the study participants used it for the treatment of severe acne cases, 24.1% for mild acne cases, and 4.1% for the treatment of rosacea skin disorder while the rest were administered it for the relieve of previous acne scars; Figure 1.

As shown in Table 2, the majority of the study patients used oral isotretinoin treatment properly in terms of nonpharmacological measures and duration of treatment use. Except for performing regular lipid profile and liver enzyme testing, around half of the study participants did not perform regular monitoring. Moreover, desperately, 58.1% of the participants did not refer back to their medical specialists when experiencing any treatment side effects. Moreover, interestingly, 20% share their treatment with family or friends, while 3.9% use expired products.

A practice score was calculated for every participant about the appropriate use of isotretinoin oral treatment. Each question was given one mark; total number of questions was 16. For the study participants, the minimum score was 2 out of 16 while the maximum score was 14 out of 16. The average score for all participants was 9.98 ± 3.01. Our results as detailed in Table 3 show statistical significance with regard to the proper use score between female and male participants where female participants got a higher score than male participants (*p* value <0.05, unpaired *T*-test). However, there was no statistical significance between different age groups or education levels and the appropriate use, although participants aged 41 years and older and those with bachelor's degrees achieved the highest average scores (*p* value >0.05, ANOVA).

Additionally, participants were asked to evaluate their knowledge about the risk of isotretinoin oral treatment on a Likert scale from 1 to 5; 1 was the least while 5 was the highest. A statistically significant result was found between the risk score and the proper use score (*p* value <0.05, ANOVA). Notably, participants who evaluated their knowledge about the risk of oral isotretinoin with the highest score group were actually using it most properly and got the highest proper use score (11.20 ± 2.53). Further details can be found in Table 3.

Participants were asked if they experienced uncommon side effects while using oral isotretinoin treatment. Out of the study participants, 11.5% experienced nausea or stomach upset, 10.5% suffered from episodes of irregular heartbeat, 7.0% had episodes of irregular heartbeats while only 3.2% were diagnosed with pancreatitis while on isotretinoin treatment. Detailed frequencies and percentages are mentioned in Table 4. Among the thirty-two married female patients that participated in the study, 96.9% did not administered oral isotretinoin during pregnancy or breastfeeding periods; however, 3.1% stated that they have administered it during breastfeeding.

With regard to participant knowledge about the risk of the misuse of isotretinoin oral treatment, 57.7% of the study participants had knowledge about its teratogenicity, 52.6% recognized its association with liver damage, and only 37.2% had knowledge about its effect on the lipid profile. Desperately, 25% of the participants think that oral isotretinoin treatment is not associated with any side effects.

## 4. Discussion

According to the Global Burden of Disease study, acne vulgaris (AV) affects roughly 85% of people between the ages of 12 and 25 [17]. This finding explains our results, as the highest usage of oral isotretinoin was observed in participants aged 19 to 25 years old. Oral isotretinoin treatment is widely recognized in published literature and clinical practice as the most commonly used treatment for moderate-to-severe acne vulgaris. However, it is associated with several common side effects that patients should be aware of, and these side effects can be less severe and occur less frequently with proper use [18]. Therefore, this study is the first in Jordan that aims to assess patient's use or misuse of oral isotretinoin besides their knowledge about the risk of the treatment side effects and the appropriate application of nonpharmacological precautionary measures.

Upon investigating the pattern of appropriate use of oral isotretinoin treatment, part of the questions of the survey was related to the correct application of nonpharmacological measures besides adherence to the prescribed treatment. Interestingly, the majority of the study participants were applying nonpharmacological measures such as eye and lip lubricant, sun protection, and skin moisturizers. This result can be justified by the good knowledge and awareness of the patient's resident in Jordan about the most common side effects of oral isotretinoin and the appropriate measures that should be applied. Based on recent research about the patient's knowledge of isotretinoin therapy in Jordan, the most recognized side effect of isotretinoin therapy was dryness (98.1%), and the study patients showed good knowledge about isotretinoin use [18].

Regarding the pattern of use of isotretinoin in Jordan, this study finds that the majority of patients are using their medication properly, with an average appropriate use score of 9.98 out of 16. This level of practice may be considered good to very good in Jordan regarding the correct use of oral isotretinoin. However, we cannot confirm the absence of misuse pattern in this study. Findings from this study present several misuse behaviours recorded by the participants including nonadherence to neither the prescribed dose nor the treatment duration, sharing their medication with family and friends or applying laser hair removal sessions while on treatment. Moreover, it is noteworthy to mention that some of the recorded behaviours are very essential for correct medication use, although they are not performed by the majority of the participants; such example is the use of the medication without medical prescription or using expired products.

Another concerning result was the high percentage of patients who did not refer back to any healthcare provider when experiencing any of the common or uncommon side effects. Alarmingly, 58% of the study participants did not refer to any medical specialist upon experiencing any of the side effects. A previously published study report stated that 1 in 8 patients do not use their referral to medical specialists the fact that will let those patients not receiving adequate care [18]. This highlights the need for patient education on the importance of consulting a healthcare provider when experiencing side effects to increase patient safety and reduce the occurrence of unwanted side effects.

A decline in the rate of performing lipid profile and liver enzyme testing preuse and during treatment is noted in this study's results. As can be noticed, it declined from 76.1% to 54.8%. This could be attributed to the poor knowledge of patients about the importance of such blood testing or to the decline in the referral rate after starting the treatment. The ideal approach to laboratory monitoring is still unclear, and there is significant variation in clinical practice [19]. Some researchers support the fact that monthly monitoring of lipid profile and liver enzymes during systematic isotretinoin treatment is not highly recommended [20, 21] while others emphasize the beneficial effect of such monitoring on patient current and future health status [22, 23].

Based on this study result and the most recent clinical practice in Jordan, most of the patients were using isotretinoin for the treatment of moderate-to-severe acne or acne scar. Interestingly, around 4% of the patients state their usage of oral isotretinoin for the treatment of rosacea skin disorder. Based on previously published research, low dose of isotretinoin can be recommended for the treatment of rosacea skin disorder which is a chronic inflammatory skin disease mostly affecting the face and characterized by recurrent erythema and flushing [24, 25].

A minority of the study participants experienced uncommon side effects of systemic isotretinoin treatments such as shortness of breath, irregular heartbeats, chest pain, and abdominal pain or were even diagnosed with pancreatitis. Those findings are also found in case reports published in the literature about medical cases of patients who suffered from the former side effects during their treatment period [26, 27]. Regarding patients' knowledge about systemic isotretinoin risk, the majority of the patients were aware about the risk of teratogenicity followed by liver damage and lipid profile abnormality. On the contrary, about 25% of the patients think there is no risk associated with it. This result is concerning, as it raises questions about the awareness of individuals residing in Jordan regarding the side effects and risks associated with oral isotretinoin treatment.

Based on the result of this descriptive study, it is important to focus on patients' practice regarding the appropriate systemic isotretinoin use. As shown, patients' appropriate use of isotretinoin was good to very good. It shows high patient discipline and awareness in specific areas such as nonpharmacological measures, patient adherence to the prescribed dose, and duration of treatment while other aspects raise attention about common misuse of isotretinoin in Jordan, such as administering the treatment without prescription, low referral percentage, and blood monitoring during treatment.

## 5. Conclusion

This cross-sectional study revealed a high percentage of oral isotretinoin use among youth in Jordan. While most patients presented good knowledge and adherence to treatment, misuse practices and lack of awareness were as desperately found among users. In conclusion, this study provides perceptions about patient practices and behaviours concerning oral isotretinoin use in Jordan, highlighting areas of both adherence and misuse and demanding directed patient education and improved monitoring practices.

### 5.1. Study Strengths and Limitations

The study on isotretinoin use among young people in Jordan exhibits several strengths, including its relevance to public health, methodological rigor in employing a cross-sectional design, comprehensive assessment of various facets of isotretinoin utilization, and implications for targeted interventions. However, limitations such as sampling bias from clinic-based recruitment, reliance on self-reported data susceptible to biases, limited generalizability beyond the Jordanian context, and the cross-sectional nature of the study temper the interpretation of findings, underscoring the need for cautious consideration and further research to validate and extend these insights.

The findings of this study underscore the importance of active actions in clinical practice regarding isotretinoin use among young patients in Jordan. Healthcare professionals should initiate widespread patient education to ensure awareness of isotretinoin risks and encourage adherence to treatment guidelines. Focus on the importance of seeking medical assistance promptly in case of side effects and regular monitoring of blood parameters during treatment should be highlighted. Additionally, healthcare providers need to be engaged in open discussions through awareness campaigns with patients to address any misconceptions or concerns they may have regarding isotretinoin therapy. Applying these approaches can enhance patient safety, optimize treatment outcomes, and lessen the possible misuse of isotretinoin in clinical practice.

## Figures and Tables

**Table 1:** Demographic data.

Demographic data of the study sample (*N* = 373)	
Parameter	Frequency (percentage %)
Nationality	
Jordanian	333 (89.3%)
Non-Jordanian	40 (10.7%)
Gender	
Female	285 (76.4%)
Male	85 (22.8%)
Age (years)	
14–18	12 (3.2%)
19–25	139 (37.3%)
26–30	68 (18.2%)
31–40	74 (19.8%)
≥41	77 (20.6%)
Region of residency	
Middle Jordan	309 (82.8%)
North Jordan	5 (1.3%)
South Jordan	19 (5.1%)
Educational level	
High school	29 (7.8%)
Diploma degree	50 (13.4%)
Bachelor's degree	254 (68.1%)
Postgraduate degree	37 (9.9%)

**Table 2:** Patients' practice about appropriate isotretinoin use.

Attitude item	Yes^*∗*^	No
Use of eye lubricant	90 (78.9%)	28 (23.7)
Use of sun protection products	128 (93.4%)	9 (6.6%)
Use of lip lubricants	123 (91.1%)	12 (8.9%)
Use of skin moisturizers	94 (68.8%)	43 (31.3%)
Avoid lazier skin cessions during treatment course	104 (86.7%)	16 (13.3%)
Avoid using isotretinoin for less time than prescribed	105 (72.9%)	39 (27.1%)
Avoid using isotretinoin for more time than prescribed	129 (83.2%)	26 (16.8%)
Avoid using higher isotretinoin dose than prescribed	112 (93.3%)	8 (6.7%)
Avoid using lower isotretinoin dose than prescribed	99 (82.5%)	21 (17.5%)
Perform lipid profile and liver enzyme testing preuse	118 (76.1%)	37 (23.9%)
Perform lipid profile and liver enzyme testing regularly during use	85 (54.8%)	70 (45.2%)
Perform vitamin A allergy testing preuse	21 (13.5%)	134 (86.5%)
Do not share treatment with family or friends	124 (80%)	31 (20%)
Do not use expired isotretinoin products	149 (96.1%)	6 (3.9%)
Got isotretinoin by medical prescription only	128 (82.6%)	27 (17.4%)
Talk to a healthcare provider about side effects	65 (41.9%)	90 (58.1%)

^*∗*^Yes is the appropriate use.

**Table 3:** Association between sociodemographics and the total proper use score.

Characteristics	Mean ± SD^*∗*^	*p* value^*∗*^
Gender		
Male	6.00 ± 1.78	<0.05
Female	11.14 ± 2.19	
Age		
14–18	9.78 ± 2.77	>0.05
19–25	9.81 ± 3.12	
26–30	9.95 ± 2.8	
31–40	10.05 ± 3.29	
≥41	11.18 ± 2.96	
Education level		
High school	8.91 ± 3.91	>0.05
Diploma degree	9.93 ± 2.58	
Bachelor degree	10.22 ± 2.85	
Postgraduate degree	8.85 ± 3.87	
Participants' risk evaluation rank^*∗∗*^		
1	8.74 ± 3.17	<0.05
2	8.83 ± 3.01	
3	9.32 ± 3.01	
4	10.73 ± 2.84	
5	11.20 ± 2.53	

^*∗*^One-way analysis of variance (ANOVA) and independent *T*-test were applied where applicable, *p* value less than 0.05 is considered significant. ^*∗∗*^Participants' risk evaluation rank from 1 to 5 where 1 is lowest risk and 5 is highest risk.

**Table 4:** Uncommon side effects experienced by the study participants.

Symptom	Yes	No
Episodes of shortness of breath	26 (7.0%)	129 (58.4%)
Episodes of irregular heartbeat	39 (10.5%)	116 (31.1%)
Nausea or stomach upset	43 (11.5%)	112 (30.0%)
Chest pain	10 (2.7%)	145 (38.9%)
Abdominal pain	22 (5.9%)	133 (35.7%)
Pancreatitis	12 (3.2%)	143 (38.3%)

## Data Availability

Data will be available on demand.
